# Estimation of Distribution Algorithm for Resource Allocation in Green Cooperative Cognitive Radio Sensor Networks

**DOI:** 10.3390/s130404884

**Published:** 2013-04-12

**Authors:** Muhammad Naeem, Udit Pareek, Daniel C. Lee, Alagan Anpalagan

**Affiliations:** 1 Department of Electrical and Computer Engineering, Ryerson University, 350 Victoria Street, Toronto, ON M5B 2K3, Canada; E-Mail: muhammadnaeem@gmail.com; 2 School of Engineering Science, Simon Fraser University, Burnaby, BC V5A 1S6, Canada; E-Mails: upareek@sfu.ca (U.P.); dchlee@sfu.ca (D.L.)

**Keywords:** green resource allocation, carbon foot print, cognitive radio, cooperative communication, estimation-of-distribution algorithm

## Abstract

Due to the rapid increase in the usage and demand of wireless sensor networks (WSN), the limited frequency spectrum available for WSN applications will be extremely crowded in the near future. More sensor devices also mean more recharging/replacement of batteries, which will cause significant impact on the global carbon footprint. In this paper, we propose a relay-assisted cognitive radio sensor network (CRSN) that allocates communication resources in an environmentally friendly manner. We use shared band amplify and forward relaying for cooperative communication in the proposed CRSN. We present a multi-objective optimization architecture for resource allocation in a green cooperative cognitive radio sensor network (GC-CRSN). The proposed multi-objective framework jointly performs relay assignment and power allocation in GC-CRSN, while optimizing two conflicting objectives. The first objective is to maximize the total throughput, and the second objective is to minimize the total transmission power of CRSN. The proposed relay assignment and power allocation problem is a non-convex mixed-integer non-linear optimization problem (NC-MINLP), which is generally non-deterministic polynomial-time (NP)-hard. We introduce a hybrid heuristic algorithm for this problem. The hybrid heuristic includes an estimation-of-distribution algorithm (EDA) for performing power allocation and iterative greedy schemes for constraint satisfaction and relay assignment. We analyze the throughput and power consumption tradeoff in GC-CRSN. A detailed analysis of the performance of the proposed algorithm is presented with the simulation results.

## 1. Introduction

Wireless sensor networks (WSN) play an important role in many industrial [1], health [2] and body monitoring systems [3,4], seismic vibration sensing [5], ad-hoc systems [6] and spectrum sensing [7] applications. Almost all modern applications and services require some form of sensors. Due to the rapid increase in WSN applications and services, in the future, the limited frequency spectrum available for WSN applications will be extremely crowded [8]. With the rapid growth and dense deployment of WSN, in the field of information and communication technologies (ICTs) they play a significant role on the global environment [9]. According to the International Telecommunication Union report [10], the primary sources of greenhouse gases are electricity generation, transport vehicles, buildings, electronic waste (e.g., batteries, small electric cells, *etc.*) and agricultural by-products. A vast portion of electricity is generated with thermal- or coal-operated turbines. The process of electricity generation is a major contributor to the emissions of green house gases. With the rapid growth and demand of WSN, future WSNs' will face three major challenges: (1) the wireless spectrum availability, (2) the demand for high data rate transmission and (3) the reduction in greenhouse gases to cope with global warming.

**Figure 1.**
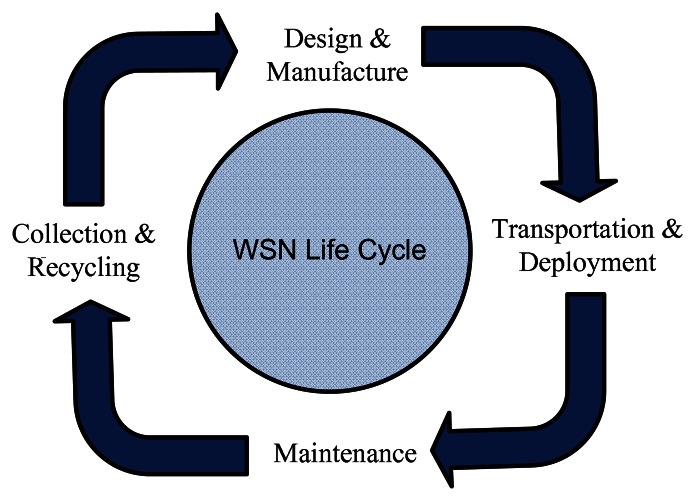
WSN life cycle.

A large portion of green house gases is composed of carbon dioxide (CO_2_) emissions. For WSN, the term *green* refers to an energy-efficient and a low carbon deployment and operation. For reducing green house gases, particularly carbon dioxide emissions, an energy-efficient resource allocation plays a significant role and has a direct impact on the lifecycle of WSN. The lifecycle of WSN is shown in Figure 1. WSN are composed of the following phases: sensor network design, manufacturing, transportation, deployment and recycling. Each phase has its role in global warming—e.g., sensor network design and the manufacturing phase require highly sophisticated computing machines that not only use ample amounts of electricity, but also produce electronic waste. Both the generation of electricity and electronic waste has a direct impact on global warming. The sensor network application, e.g., multiple tracking, traffic monitoring, spectrum sensing or any other commercial/military application, requires a dense deployment of sensors. In the deployment, maintenance and recycling phases, the use of transportation is the major source of global warming.

An energy efficient-design of WSN can reduce its contribution towards global warming. Research in green ICTs will enable the wireless system designers to develop and design cellular, ad-hoc and WSN systems that will achieve user data rate demands with minimum power usage and, thus, contribute to reduction of green houses gases [11]. A combination of the intelligent design of future WSN with emerging wireless technologies, such as cooperative communication and cognitive radio, can help in coping with the crowded spectrum, the demand for a high data rate and global warming issues. Cognitive radio is an interesting concept for solving the problem of spectrum availability by reusing the under-utilized licensed frequency bands [12]. Formally, a cognitive radio is defined as [12], “a radio that changes its transmitter parameters based on the interaction with its environment”. The cognitive radio has been mainly proposed to improve the spectrum utilization by allowing (unlicensed) secondary users (SUs) to use under-utilized licensed frequency bands. The IEEE 802.22 standard for Wireless Regional Area Network (WRAN) addresses the cognitive radio technology by allowing access to white spaces in the licensed TV band. In North America, the frequency range for the IEEE 802.22 standard will be 54–862 MHz, while the 41–910 MHz band will be used in the international standard. In cognitive radio networks, licensed users and unlicensed users are known as primary and secondary users, respectively. In [13–20], the authors use cognitive radio technology for wireless sensor networks. A detailed architecture, topologies and potential applications of cognitive radio sensor networks (CRSNs) are presented in [17]. The potential applications include indoor sensing, multimedia, multi-class heterogeneous sensing, body area networks and real-time surveillance.

In the context of environmentally friendly cognitive radio sensor network, cooperative communication can help in reducing the total transmission power and, thus, reducing the CO_2_ emissions. The relays play an important role in many real-life wireless sensor network applications [21–26]. Experimental results and WSN testbeds for cooperative communication also provide insight into the effect of relays on the WSN lifetime. In [21], the authors experimentally show the performance of the amplify and forward scheme in an orthogonal frequency-division multiplexing (OFDM)-based system. The results show that the amplify and forward scheme is highly beneficial for power-aware wireless sensor networks. In [22,23], the authors investigate the effect of relays in terrestrial and underground WSN. In a terrestrial WSN, reliable communication in a dense environment is very important. Terrestrial sensor nodes must be able to effectively communicate data back to the base station. Underground WSNs consist of a number of sensor nodes buried underground or in a cave or mine used to monitor underground conditions. Additional sink nodes are located above ground to relay information from the sensor nodes to the base station. The authors show that with limited battery power (which cannot be recharged, due to geographical constraint) in terrestrial and underground sensor nodes, energy (battery life) can be conserved with the use of relays, a short transmission range, in-network data aggregation, eliminating data redundancy, minimizing delays and using low duty-cycle operations. A dual-hop energy-efficient cooperative spectrum sensing scheme with amplify-and-forward relaying in CRSN is proposed in [7]. In [4], the authors presented a relay-assisted human monitoring system in a body area network that uses 802.15.3/802.15.4 for its monitoring application. The performance analysis of dual-hop relaying in CRSN is described in [27]. In [28], the authors presented spectrum sensing and communication protocols for a dual-hop sensor relay network operating in the VHF-UHF band. Information theoretic data gathering and the effect of relaying in CRSN are described in [29]. In [30], a cognitive dual-hop relaying base sensing-transmission protocol is proposed. In [31], the authors presented an optimal solution for source-sum-power minimization in multi-sensor single-relay networks. Subspace-based cooperative spectrum sensing and correlation-based sensing for CRSN were proposed in [18] and [32], respectively.

A relay-assisted WSN for volcanic monitoring is investigated in [24]. The challenges of a WSN application for volcanic data collection include reliable event detection, efficient data collection, high data rates and sparse deployment of nodes. In the proposed WSN, each sensor node is a T-mote sky device equipped with an external omni-directional antenna, a seismometer, a microphone and a custom hardware interface board. Some of the sensor nodes are equipped with a single axis Geospace Industrial GS-11 Geophone with a corner frequency of 4.5 Hz, while the other two sensor nodes carried triaxial Geospace Industries GS-1 seismometers with corner frequencies of 1 Hz. The custom hardware interface board was designed with four Texas Instruments AD7710 analog to digital converters to integrate with the T-mote sky devices. Each sensor node draws power from a pair of alkaline D-cell batteries. Sensor nodes are placed approximately 200 to 400 meters apart from each other. Sensor nodes relay data to a gateway node. The gateway node, connected to a long-distance Free- Wave radio modem, transmits the collected data to the base station. The authors inspected the data for three weeks and observed that the network sensed 230 eruptions and other volcanic events. The authors also investigated the performance of relays in volcanic events. A three-level wireless sensor network for oil well health monitoring is proposed in [26]. Relays are used to transfer the data from one level to the other levels. An amplify and forward base linear WSN to increase the coverage is proposed in [25]. In [33], the authors present relay scheduling in a time-slotted source relay destination system, where a sensor (the source) has the option to have another sensor (the relay) help transmit its data to the destination. From an energy efficiency perspective, it is shown by the authors that the source may achieve the same bit error rate (BER) for a lower transmission power if it uses a relay, as compared to a direct transmission.

In sensor networks, the transmission power dissipated by a sender node to transmit each bit of data to a receiver node is directly dependent on the distance between them. This use of multi-hop communication may reduce overall energy consumption; some nodes can be overloaded and drain out their energy more quickly (and die), as compared to some other nodes in the network. This may produce an undesirable effect on the functionality of the networks, even causing the network to become inoperable. The use of multiple relays that convey the same data with low power can reduce the chances of WSN failure. The major challenge is how to assign multiple relays efficiently that will increase the throughput of WSN and reduce the power consumption. One open research question of CRSN is the per-hop throughput optimization. Increasing the number of hops will increase the delay, complexity, deployment and transportation cost [34].

### 1.1. Contributions and Organization

In this paper, we investigate dual-hop CRSN that jointly maximizes the throughput and minimizes the total transmission power by assigning multiple relays to the users. In the future, we will investigate the optimal number of hops in CRSN to jointly maximize the throughput and minimize the total transmission power (or minimize the CO_2_ emissions). We use a combination of shared-band non-regenerative amplify and forward relaying and cognitive radio to solve the problem of the crowded spectrum, the demand for a high data rate and global warming.

Data rate maximization and power minimization are two conflicting objectives. Determining the optimal set of decision variables for a single objective, e.g., CO_2_ emissions minimization, can result in a non-optimal set with respect to other objectives, e.g., sum-capacity (throughput) maximization. In our formulation, we use the normalized weighted sum method (WSM) to combine these conflicting objectives. In WSM, the weight of each objective is proportional to its importance, placed for decision-making. A WSM [35] without normalization would result in a biased fitness function—e.g., if the value of one objective function is in the range [0, 1] and the value of second objective is in the range [0, x] (where 1 < *x* ≤ ∞), then the second objective produces bias in the weighted fitness function. In this work, we normalize all the objective values within the range [0, 1]. We formulate our problem in a way that the range of the combined objective function is always within 0 and 1.

According to the best knowledge of the authors, there is no joint multiple relay assignment and power allocation scheme in the literature that deals with the analysis and optimization of the energy efficiency in a shared band multi-user cognitive radio system. The motivation of this work is to fill the gap, especially important for future green radio communications, with the aim of analyzing the shared-band multiple relay assignment and power allocation problem that maximizes the data rate and minimizes the CO_2_ emissions. The main contributions of this paper are summarized as follows:

1. We propose a multi-objective optimization framework that jointly exploits the crowded spectrum, the demand for a high data rate and global warming with the help of relay-assisted GC-CRSN. The proposed multi-objective framework jointly performs multiple relay assignment and power allocation in GC-CRSN, while optimizing two conflicting objectives. The first objective is to maximize the total throughput, and the second objective is to minimize the total transmission power of GC-CRSN.
2. For multiple relay assignment, we use a shared-band amplify and forward protocol. We also drive an upper bound on the data rate of the shared band amplify and forward protocol. This upper bound is useful for normalization in multi-objective optimization.
3. The proposed joint multiple relay assignment and power allocation problem is a non-convex mixed-integer non-linear optimization problem (NC-MINLP), which is generally NP-hard. We introduce a hybrid heuristic algorithm for this problem. The hybrid heuristic is a combination of the estimation-of-distribution algorithm (EDA) for performing power allocation and an iterative greedy algorithm for constraint satisfaction and relay assignment.
4. In addition to applying the EDA to the constrained multi-objective optimization problem for GC-CRSN, we also propose a modification in the EDA that greatly improves its performance.
5. A detailed analysis of the performance of the proposed algorithm is presented with the simulation results.

We use ***A***, ***a*** and *a* to represent matrix, vector and an element of a vector, respectively. When *a_i_* ≥ 0 for all components, *i*, of a vector, *a*, we use *a* ≥ 0. Table 1 presents the summary of notations and symbols used in this paper. The rest of the paper is organized as follows. The system model is presented in Section 2. In Section 3, we present our EDA and its improved version. Simulation results are presented in Section 4.

## 2. System Model and Problem Formulation

We consider a two-hop wireless sensor network with one transmitter node (source), *K* receiver nodes (also known as secondary users/nodes), *L* relay nodes and *M* primary users/nodes. Each relay, transmitter, and receiver is equipped with a single antenna. We denote by $h_{l}^{s}$, the channel from the source to the *l*th relay, $h_{k}^{l}$ , the channel from the *l*th relay to the *k*th secondary user, $g_{m,k}^{s}$, the channel from the source to the *m*th primary user, and $g_{m,k}^{l}$, the channel from the *l*th relay to the *m*th primary user. We denote by p*_l_*, the *l*th relay's transmission power; $p_{l}^{\text{max}}$ is the maximum power of the *l*th relay, $p_{k}^{s}$, the source power in the *k*th user band, and *P_s_*, the maximum source power, *i.e.*, $\sum_{k}p_{k}^{s}\leq P_{s}$. In our system model, each user will receive the data on a separate frequency band. Each relay will transmit and receive in the same frequency band.

We consider a half-duplex shared band amplify and forward (AF) protocol in which each symbol is transmitted in two time slots; in the first time slot, by the source, and in the second time slot, by the relays. In the first time slot, the signal received by the lth relay (after listening to the *k*th SU's band) can be written as $\sqrt{p_{k}^{s}}h_{l}^{s}s+Z_{l}$, where complex-valued *s* represents the transmitted symbol and *Z_l_* represents the complex-valued white Gaussian noise at the *l*th relay. The symbol value, *s*, *E*(|*s*|^2^) = 1 and $\frac{N_{o}}{2}$ are the power spectral density of the noise *Z_l_*. The noise power, *N*, in watts, in each SU band can be written as $N=\left(\frac{N_{o}}{2}\right)2W$, where *W* is the bandwidth of each SU band [36]. In the second time slot, the relays amplify the received signal and re-transmit the amplified signal. The channel capacity of the *k*th user for shared band, AF, is [37]:

(1) $$C_{k}=\frac{1}{2}\log\;\left[1+\frac{p_{k}^{s}}{N}\left({\left|h_{k}^{s}\right|}^{2}+\Omega_{k}\right)\right]$$

where $\Omega_{k}=\left(\frac{{\left(\sum_{l=1}^{L}\left|h_{l}^{s}h_{k}^{l}\right|\beta_{l}\sqrt{p_{l}}\right)}^{2}}{1+\sum_{l=1}^{L}{\left(\left|h_{k}^{l}\right|\beta_{l}\sqrt{p_{l}}\right)}^{2}}\right)$ and $\beta_{l}={\left(\sqrt{p_{k}^{s}{\left|h_{l}^{s}\right|}^{2}+\frac{N}{2}}\right)}^{-1}$. Note that the capacity formula for the shared band, AF, is not a concave function of the relay powers. This is due to the term Ω_k_. We define as a binary assignment indicator:

$$\varepsilon_{k}^{\text{l}}=\left\{ \begin{array}{ll} 1 & \text{if the}\;l\text{th relay is assigned to the}\;k\text{th user}\\ 0 & \text{otherwise} \end{array}\right.$$

The channel capacity of the *k*th user for AF relaying with the binary assignment indicator is:

(2) $$C_{k}^{\varepsilon }=\frac{1}{2}\log\;\left[1+\frac{p_{k}^{s}}{N}\left({\left|h_{k}^{s}\right|}^{2}+\Omega_{k}^{\varepsilon }\right)\right]$$

where $\Omega_{k}^{\varepsilon }=\left(\frac{{\left(\sum_{l=1}^{L}\varepsilon_{k}^{l}\left|h_{l}^{s}h_{k}^{l}\right|\beta_{l}\sqrt{p_{l}}\right)}^{2}}{1+\sum_{l=1}^{L}\varepsilon_{k}^{l}{\left(\left|h_{k}^{l}\right|\beta_{l}\sqrt{p_{l}}\right)}^{2}}\right)$.

Our first objective is to maximize the sum-rate capacity, $\sum_{k=1}^{K}C_{k}$. To normalize the first objective between 0 and 1, we will divide the sum-rate capacity with its upper bound, $\sum_{k=1}^{K}C_{k}^{\text{max}}$.

**Lemma 1.** *The ecision variable-free upper bound of* Equation (1) is $C_{k}^{\text{max}}=\frac{1}{2}\log\left[1+\frac{P_{s}}{N}\left({\left|h_{k}^{s}\right|}^{2}+\sum_{l}{\left|h_{l}^{s}\right|}^{2}\right)\right]$.

*Proof.* It is easy to see that *C_k_* in Equation (1) is an increasing function of the source power. We can set the source power to its maximum transmission power, *P_s_*. We will get an upper bound as:

(3) $$\begin{array}{ll} C_{k} & =\frac{1}{2}\log\;\left[1+\frac{p_{k}^{s}}{N}\;\left({\left|h_{k}^{s}\right|}^{2}+\frac{{\left(\sum_{l}\left|h_{l}^{s}h_{k}^{l}\right|\beta_{l}\sqrt{p_{l}}\right)}^{2}}{1+\sum_{l}{\left(\left|h_{k}^{l}\right|\beta_{l}\sqrt{p_{l}}\right)}^{2}}\right)\right]\\ & <\frac{1}{2}\log\;\left[1+\frac{P_{s}}{N}\;\left({\left|h_{k}^{s}\right|}^{2}+\frac{{\left(\sum_{l}\left|h_{l}^{s}h_{k}^{l}\right|\beta_{l}\sqrt{p_{l}}\right)}^{2}}{\sum_{l}{\left(\left|h_{l}^{s}\right|\beta_{l}\sqrt{p_{l}}\right)}^{2}}\right)\right] \end{array}$$

Applying Cauchy-Schwartz inequality, we will get:

(4) $$\begin{array}{l} \leq \frac{1}{2}\log\;\left[1+\frac{P_{s}}{N}\;\left({\left|h_{k}^{s}\right|}^{2}+\frac{\sum_{l}{\left|h_{l}^{s}\right|}^{2}\sum_{l}{\left(\left|h_{k}^{l}\right|\beta_{l}\sqrt{p_{l}}\right)}^{2}}{\sum_{l}{\left(\left|h_{k}^{l}\right|\beta_{l}\sqrt{p_{l}}\right)}^{2}}\right)\right]\\ =\frac{1}{2}\log\;\left[1+\frac{P_{s}}{N}\;({\left|h_{k}^{s}\right|}^{2}+\sum_{l}{\left|h_{l}^{s}\right|}^{2})\right] \end{array}$$

Mathematically, we can write the normalized sum-rate as:

(5) $$F_{1}=\frac{\sum_{k=1}^{K}C_{k}}{\sum_{k=1}^{K}C_{k}^{\text{max}}}$$

The second objective is to reduce the carbon footprint or CO_2_ emissions. The CO_2_ emissions are measured in grams. If *P* is the power used in the transmission and *X* is a constant in grams/watt, then the product of *P* and *X* (*i.e.*, *PX)* represents the CO_2_ emissions in grams. The value of *X* is different for different types of material (fuel) used for electricity generation. There are three major sources of fuel for electricity generation. These fuels are oil, gas and coal. The value of *X* for lignite/brown coal, natural gas, crude oil and diesel oil is 940, 370, 640 and 670 grams/watt, respectively [11]. We define by $E_{l}^{CO_{2}}=X\;p_{l}$, the CO_2_ emissions due to the *l*th relay, and $E_{s,k}^{CO_{2}}=X\;p_{k}^{s}$, the CO_2_ emissions due to the source transmission power in the *k*th user band. We will normalize the CO_2_ emissions objective function with $E_{l^{\text{max}}}^{CO_{2}}+E_{s^{\text{max}}}^{CO_{2}}$, where $E_{l^{\text{max}}}^{CO_{2}}=\sum_{l}Xp_{l}^{\text{max}}$ and $E_{s^{\text{max}}}^{CO_{2}}=XP_{s}$. We can write the objective of CO_2_ emissions as:

(6) $$F_{2}=\frac{E^{CO_{2}}}{E_{\text{max}}^{CO_{2}}}$$

where $E^{CO_{2}}=\sum_{l}E_{l}^{CO_{2}}+\sum_{k}E_{s,k}^{CO_{2}}$ and $E_{\text{max}}^{CO_{2}}=E_{l^{\text{max}}}^{CO_{2}}+E_{s^{\text{max}}}^{CO_{2}}$.

The joint objective of the GC-CRSN problem is to maximize the data-rate—*i.e.*, *F*_1_—and minimize the CO_2_ emissions—*i.e.*, *F*_2_. For joint optimization, we need to transform the objectives, *F*_1_ and *F*_2_, into a joint minimization (or maximization) objective. Since both objectives are normalized and bounded between 0 and 1, we can make the joint minimization objective as:

(7) $$F=w_{1}\left(1-F_{1}\right)+w_{2}F_{2}$$

Using Equation (7), we can write the multi-objective optimization for GC-CRSN as:

(8) $$\begin{array}{ll} \begin{array}{c} OP1:\\ \underset{\varepsilon ,p_{l},p_{s}}{\min}\\ \text{subject to} \end{array} & F\\ & \begin{array}{l} C1:\sum_{k}^{K}\varepsilon_{l}^{k}\leq 1,\forall \;l\\ C2:\sum_{k=1}^{K}p_{k}^{s}\leq P_{s}\\ C3:0\leq p_{l}\leq \sum_{k=1}^{K}\varepsilon_{l}^{k}p_{l}^{\text{max}},\forall \;l\\ C4:p_{k}^{s}{\left|g_{m,k}^{s}\right|}^{2}\leq I_{m,k}^{\max},\forall \left(m,k\right)\\ C5:\sum_{l=1}^{L}\varepsilon_{l}^{k}p_{l}{\left|g_{m,k}^{l}\right|}^{2}\leq I_{m,k}^{\max},\forall \left(m,k\right)\\ C6:\varepsilon_{l}^{k}\in \left\{0,1\right\} \end{array} \end{array}$$

In *OP*1, the constraint, *C*1, assures that a relay can only be assigned to one secondary user; *C*2 and *C*3 are the power constraints. The constraints, *C*3, ensures that if the *l*th relay is not assigned to any secondary user, then the transmission power of the lth relay should be zero. Constraints, *C*4 and *C*5, are the interference constraints. The objective function in *OP*1 is bounded by zero and one. The formulation in *OP*1 is a multi-objective non-convex mixed-integer non-linear programming problem, which is generally NP-hard. In the next section, we will present a low-complexity hybrid estimation-of-distribution algorithm (EDA) for the GC-CRSN multi-objective optimization problem.


**Algorithm 1** Pseudo code for a typical EDA.
1:*Initialize the population with uniform probability distribution*2:**while** (true) **do**3: *Evaluate the population*4: *Rank the population according to fitness*5: *Select the best individuals*6: *Estimate the probability distribution from best selected individuals*7: *Sample the probability model to generate new population*8: **if** Termination Criterion Satisfied **then**9:  break10: **end if**11:**end while**


## 3. Hybrid EDA Solution for the GC-CRSN Problem

In this section, we will present a hybrid scheme to solve the GC-CRSN multi-objective problem as given in *OP*1. The proposed scheme is a combination of an evolutionary estimation-of-distribution algorithm for power allocation and an iterative greedy scheme for relay assignment. The iterative scheme also ensures the feasibility of the optimization solution.

Evolutionary algorithms (EAs) in general have been often used to solve multi-objective optimization problems. EAs are inspired by the theory of biological evolution. The candidate solutions to a multi-objective optimization problem are represented as individuals in the population. In EAs, the objective function value of a candidate solution indicates the fitness of the individual, which is associated with the concept of natural selection [38]. Unlike other EAs, such as the genetic algorithm (GA), in EDA, the individuals are generated without the crossover and mutation operators. Instead, in EDA, a new population is generated based on a probability distribution, which is estimated from the best-selected individuals of the previous iterations [39].

Algorithm 1 presents a pseudo code of a typical evolutionary EDA. At the start of the EDA algorithm, the population is generated by sampling the uniform probability distribution. After getting the population, the algorithm evaluates each individual in the population and ranks the population according to the fitness of each individual. Then, the algorithm selects the best individual from the ranked population with the help of the probability of selection. These selected individuals are used to estimate the new probability distribution for the next iteration. The algorithm again samples the probability model to generate the new population. The algorithm continues its execution until some predefined termination criterion is satisfied. In the next section, we will describe EDA for the GC-CRSN problem.

### 3.1. EDA for GC-CRSN Problem

In the implementation of EDA for the optimization problem in *OP1*, each individual can be designated by an (*L* + *K*)-dimensional real-valued vector. First, *L*-dimensions are for relay powers, and the next *K* dimensions are for source power. We denote by Δ*_t_*, the population at the *t*th iteration, and |Δ_t_| denotes its cardinality, *η_t_*, the set of best candidate solutions selected from set |Δ_t_| at the tth iteration, and *ρ_s_* is the selection probability. The EDA selects *ρ_s_*|Δ*_t_* | individuals from the set | Δ*_t_* | to make up the set *η_t_*. In our implementation of EDA, each individual represents the transmission power of the relays and source. We denote by a row vector, *P* = (*p_1_*, *p_2_*,⋯, *p_L_*, *p_L_*_+1_,⋯, *p*_*L*__+*k*_), as an individual in the population, where *p_i_*, *i* = 1,2,⋯, *L* is the relay power vector and *p_i_*, *i* = *L* + 1, *L* + 2,⋯, *L* + *K* is the source power vector. The transmission power of the each relay and source is bounded by *W_Low_* and *W_High_*, where *W_Low_* and *W_High_* are the lower and upper limit of the EDA search window.

In each iteration, the EDA maintains a population of individuals. Population Δ*_t_* can be specified by the following | Δ_*t*_| × (*L* + *K*) matrix:

(9) $$\Lambda_{\text{EDA}}=\left(\begin{array}{c} P^{1}\\ P^{2}\\ \bar{\vdots }\\ P^{\left|\Delta_{t}\right|} \end{array}\right)=\left(\begin{array}{cccc} p_{1}^{1} & p_{2}^{1} & \cdots & p_{L+K}^{1}\\ p_{1}^{2} & p_{2}^{2} & \cdots & p_{L+K}^{2}\\ \vdots & \vdots & \vdots & \vdots \\ p_{1}^{\left|\Delta_{t}\right|} & p_{2}^{\left|\Delta_{t}\right|} & \cdots & p_{L+K}^{\left|\Delta_{t}\right|} \end{array}\right)$$

**Figure 2.**
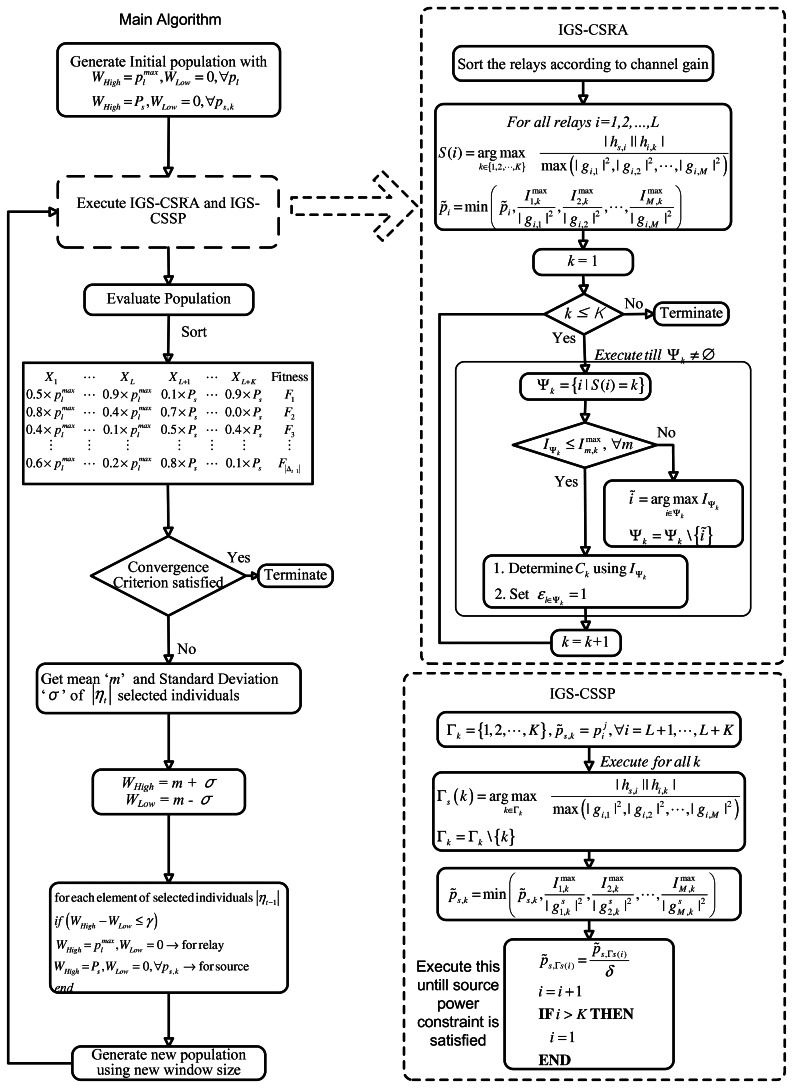
EDA flow diagram with IGS-CSRA and IGS-CSSP.

Each row of the matrix, Λ_*EDA*_, represent an individual. There are |Δ_*t*_| individuals in the population, and each individual has *L* + *K* elements. A flow diagram of the EDA algorithm is shown in Figure 2. The EDA applied to the *OP*1 problem can be described in the following steps: Step 0: Generate an initial population, Δ_0_. Each element of matrix, Λ_*EDA*_, is obtained from the following formula:

(10) $$\begin{array}{c} p_{i}^{j}=W_{\text{Low}}+\left(W_{\text{High}}-W_{\text{Low}}\right)\times \text{rand},\\ \forall i=1,2,\cdots ,L+K,\;j=1,2,\cdots ,\left|\Delta_{0}\right| \end{array}$$

where ‘rand’ is a random number generated from a uniform distribution between 0 and 1. Initially, we set *W_Low_* = 0, $W_{\text{High}}=p_{l}^{\text{max}}$ for relay powers and *W_Low_* = 0, *W_High_* = *P_s_* for the source power. For iterations, *t =* 1, 2, ., *I_Ter_*, follow Step 1 through Step 8:

Step 1: Evaluate the individuals in the current population, Δ_t−1_, according to the fitness function, f(). For *OP*1, we use the function as described in Equation (7) as the fitness function—*i.e.*, *F* = *w*_1_ (1 − *F*_1_) + *w*_2_*F*_2_. The best fitness function in any iteration is the function with the minimum value. Sort the candidate solutions (individuals in the current population) according to their fitness orders.
Step 2: Rank the candidate solutions (individuals in the current population) according to their fitness orders.
Step 3: In this step, the algorithm determines the assignment variable, $\varepsilon =\left[\varepsilon_{1}^{1},\varepsilon_{1}^{2},\cdots ,\varepsilon_{K}^{L}\right]$, for each individual heuristically. Step 1 does not guarantee a feasible solution. Feasibility check and constraint satisfaction are also performed in step 3.
  We propose an iterative greedy scheme for constraint satisfaction and relay assignment (IGS-CSRA). The IGS-CSRA ensures that the constraints C1, C3 and C5 are satisfied. We also propose an iterative algorithm for the source power such that constraints C2 and C4 are satisfied. We call this method the iterative greedy scheme for constraint satisfaction of source power (IGS-CSSP). The IGS-CSRA and IGS-CSSP are shown in Figure 2 and described in Sections 3.2 and 3.3. At the end of this step, the algorithm has a population, which comprises individuals with feasible relay and source power levels and the associated assignment variables *ε*.
Step 4: If the convergence criterion (e.g., number of iterations) is satisfied, then terminate; else, continue.
Step 5: Select the best |*η*_*t*−1_| = *ρ_s_* |Δ_*t*−1_| candidate solutions (individuals) from the current population, Δ_*t*−1_. This selected population is used to compute the mean and standard deviation of the selected individuals.
Step 6: Determine the mean, ‘m’, and standard deviation, *σ*. Based on these estimates of ‘m’ and *σ*, update the search window bounds, *W_Low_* and *W_High_*, as *W_Low_* = m-σ and *W_High_* = m+σ.
Step 7: Generate new |*η*_*t*−1_| individuals on the basis of this new estimated *W_Low_* and *W_High_* using Equation (10). Combine these newly generated |*η*_*t*−1_| individuals with members of *η*_*l*−1_ to form a new population, Δ*_l_*.
Step 8: Go to step 1 and repeat the steps.

From a practical point of view, the following observations and suggestions can help in implementing the proposed EDA for relay assignment and power allocation in a green cooperative cognitive radio sensor network. A good sensing mechanism is inevitable for a cognitive radio that will add robustness to the system. We also need optimal parameter settings of EDA for different geographical regions. A good estimate of the initial population of EDA can also increase its convergence rate towards good solutions. This increase in convergence rate eventually decreases the computational complexity of the central controller. Now, we will explain iterative greedy schemes that jointly assign relays and ensure the feasibility of the solution.

### 3.2. Iterative Greedy Scheme for Constraint Satisfaction and Relay Assignment (IGS-CSRA)

In this section, we present an iterative greedy scheme for constraint satisfaction and relay (IGS-CSRA). The IGS-CSRA scheme will be executed on each EDA individual in the population. IGS-CSRA illustrates the relay assignment for the EDA individual indexed by $j\;‐\;P^{j}\;=\;\left(p_{1}^{j},p_{2}^{j},\cdots ,p_{L+K}^{j}\right)$. We denote by ${\tilde{p}}_{i}=p_{i}^{j}$, *i* = 1,⋯, *L*, the *i*th relay's power level of the *j*th individual in the population. The proposed algorithm has two stages. In the first stage, based on the channel conditions, relays are assigned to the secondary users without satisfying the interference constraint. In the second stage, the algorithm performs final assignment under the constraint that interference to the primary users is satisfied.

For developing this greedy algorithm, we can view the product of the channel from the *i*th relay to the *k*th secondary user and the channel from the source to the *i*th relay as profit taken from investing one unit of transmission power to relay *i*. We also view the channel from the ith relay to its primary users as loss. In particular, our algorithm views max(|*g*_*i*,1_|^2^, |*g*_*i*,2_|^2^,⋯, |*g_i,M_*|^2^) as loss incurred from investing unit transmission power to relay *i*.

Stage 1: In this stage, the algorithm assigns each relay to the secondary user that gives the maximum profit to loss ratio. The profit is secondary users channel gain—*i.e.*, |*h*_*s*,*i*_| |*h*_*i*,*k*_|—and loss is the maximum channel gain from the secondary user to the primary users—*i.e.*, max(|*g*_*i*,1_|^2^, |*g*_*i*,2_|^2^,⋯, |*g_i,M_*|^2^).
  Mathematically, for each relay *i*, the algorithm temporarily assigns a secondary user:
  $$S(i)=\underset{k\in \left\{1,2,\cdots K\right\}}{\arg\max}\frac{\left|h_{s,i}\right|\left|h_{i,k}\right|}{\max\left({\left|g_{i,1}\right|}^{2},{\left|g_{i,2}\right|}^{2},\cdots ,{\left|g_{i,M}\right|}^{2}\right)}$$
  where *S* is an *L*-dimensional vector that stores this assignment. At the end of Stage 1, relays are assigned to the secondary users with the power *p_i_*˜, *i* = 1,⋯, *L*.
  Note that the relays' power levels randomly generated by the EDA algorithm can violate the constraint of limited interference to the primary users. In the next stage, the algorithm refines the relay assignment done in Stage 1 and adjusts the power level of each relay, so that interference to the primary users is satisfied.
Stage 2: At the start of the second stage, the algorithm starts adjusting the relays' power levels if there is a violation of the primary users' interference constraint. First, the algorithm examines for each relay *i* whether its transmission power would still violate any interference constraint, even if all other relays' power level were set to zero. If *p_i_*˜ violates any of the interference constraint, $I_{m,k}^{\max}$, even under the assumption that other relays' transmission power levels are all set to 0, then the algorithm first makes the following adjustment:
  $${\tilde{p}}_{i}=\min\left({\tilde{p}}_{i},\frac{I_{1,k}^{\text{max}}}{{\left|g_{i,1}\right|}^{2}},\frac{I_{2,k}^{\text{max}}}{{\left|g_{i,2}\right|}^{2}},\cdots ,\frac{I_{M,k}^{\text{max}}}{{\left|g_{i,M}\right|}^{2}}\right),\forall \left(i,k\right)$$

With this power adjustment, each user individually guarantees constraint satisfaction of the primary users' interference constraint. After the power adjustment, the algorithm iterates over the secondary users and completes the assignment of relays.

At the *k*th iteration, the algorithm determines the set of relays, Ψ*_k_*that are assigned to the *k*th secondary user in Stage 1. Then, the algorithm checks whether the relays in the set Ψ*_k_* satisfy the interference constraint at all the primary users. If the relays in the set, *Ψ_k_*, violate the interference constraint at any primary user; then the algorithm iteratively removes the relay from the set, *Ψ_k_*, that causes maximum interference to the primary users. Mathematically, this determines the relay with the highest interference from the set, *Ψ_k_*, as:

$$\tilde{i}=\underset{i\in \Psi_{k}}{\arg\max\;}I_{\Psi_{k}}$$

This relay removal process continues until the relays in the set, *Ψ_k_*, satisfy the interference constraint. When the algorithm has a set of relays that satisfy the interference constraint, then the algorithm determines the capacity of the *k*th user and it sets *ε_l∈_*Ψ*_k_* = 1.

The algorithm executes till all the secondary users get their assigned relays. In the next subsection, we will present the iterative greedy scheme and constraint satisfaction for source power.

### 3.3. Iterative Greedy Scheme and Constraint Satisfaction for Source Power (IGS-CSSP)

Now, we will describe IGS-CSSP. We denote by ${\tilde{p}}_{s,k}=p_{i}^{j}$, ∀*i* = *L*+ 1, *L*+ 2,⋯, *L* + *K*, the source power level at the *k*th user band in the *j*th sample drawn by the EDA. We denote by Γ*_s_*, a vector that will be used for user indices, Γ*_k_*, a set of users, and *δ*, a power control factor. The power control factor is used for source power adjustment. This adjustment will be done iteratively until all the interference constraints related to the source power are satisfied. The proposed IGS-CSSP is also a two-stage algorithm.

Stage 1: In the first stage, users are ranked according to their channel conditions. Similar to the IGS-CSRA, the maximum profit to loss ratio criterion is used to rank the users. The profit is secondary users channel gain, *i.e.*, |*h*_*s*,*i*_| |*h*_*i*,*k*_|, and loss is the maximum channel gain from the secondary user to the primary users, *i.e.*, max(|*g*_*i*,1_|^2^, |*g*_*i*,2_|^2^,⋯, |*g*_*i*,*M*_|^2^). Mathematically:
  $$\Gamma_{s}\left(i\right)=\underset{k\in \Gamma_{k}}{\arg\max}\frac{\left|h_{s,i}\right|\left|h_{i,k}\right|}{\max\left({\left|g_{i,1}\right|}^{2},{\left|g_{i,2}\right|}^{2},\cdots ,{\left|g_{i,M}\right|}^{2}\right)}$$
  In the next stage, the power of each user will adjusted according to their ranks.
Stage 2: At the start of the second stage, the algorithm starts adjusting the source power levels if there is a violation of the primary users' interference constraint, using the expression:
  $${\tilde{p}}_{s,k}=\min\;\left({\tilde{p}}_{s,k},\frac{I_{1,k}^{\text{max}}}{{\left|g_{s,1}\right|}^{2}},\frac{I_{2,k}^{\text{max}}}{{\left|g_{s,2}\right|}^{2}},\cdots ,\frac{I_{M,k}^{\text{max}}}{{\left|g_{s,M}\right|}^{2}}\right),\forall k$$
  After source power adjustment with the interference constraint, the algorithm verifies the power constraint. If the source power constraint is not satisfied, then the algorithm adjusts the source power using power control factor, *δ*, till the constraint is satisfied. The user with the worst channel condition is reduced first using the power control factor, *δ*. This process will be executed for all users till we get a feasible solution.

At the end of the algorithm, we shall have a feasible solution. Since, we are using the half-duplex amplify and forward protocol, both IGS-CSRA and IGS-CSSP will execute independently.

### 3.4. Modified EDA (MEDA)

During the execution of EDA, the difference between the search window bounds, *W_Low_* and *W_High_*, may diminish as the iterations proceeds. This may cause the EDA to get stuck in a local search space and result in premature convergence. The premature convergence may occur if the difference between *W_Low_* and *W_High_* diminishes to an extremely small value. In that case, at every future iteration, the algorithm will generate nearly the same power levels. In this section, we will propose a modification in the traditional EDA algorithm that can improve the EDA's performance. The modification includes the introduction of thresholds in EDA to avoid premature convergence. We name this algorithm the Modified EDA (MEDA).

We suggest restoring the *W_Low_* and *W_High_* to their initial values ( *W_Low_* = 0, $W_{\text{High}}=p_{l}^{\text{max}}$ for relay powers and *W_Low_* = 0, *W_High_*= *P_s_* for source power) when the difference between W_Low_ and *W_High_* is less than a pre-specified threshold, *γ*, *i.e.*:
**if** (*W_High_* − *W_Low_* ≤ *γ*) **then** *W_Low_* = 0,*W_High_* = 
$p_{\text{l}}^{\text{max}}$ → *for relays* *W_Low_* = 0,*W_High_* = *P_s_* → *for source***end if**

**Figure 3.**
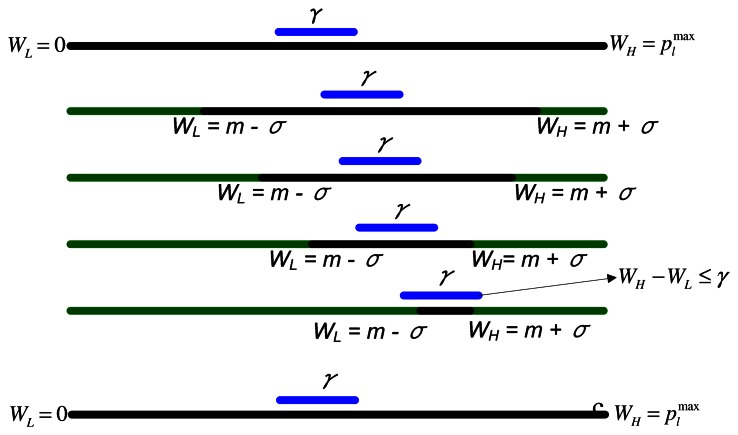
EDA thresholding.

The above steps are illustrated in Figure 3. In the next section, we present some experimental results, which show the effect of threshold on the performance of EDA.

## 4. Numerical Results

In this section, we present simulation results to demonstrate the performance of the proposed EDA and MEDA. The impact of network parameters (e.g., number of SUs, number of relays) is also investigated.

### 4.1. Simulation Setup and Common Parameters

In all the simulations, the channels between source, relays and destinations have independent complex Gaussian distribution. Some common parameter values for simulation are shown in Table 2. In all simulations, the channel gain, *h*, is modeled as [40]:

(11) $$h=\Phi K_{o}{\left(\frac{d_{o}}{d}\right)}^{\beta }$$

where *K_o_* is a constant that depends on the antenna characteristic and average channel attenuation, *d_o_* is the reference distance for the antenna far field, *d* is the distance between the transmitter and receiver, *β* is the path loss constant and Φ is the Rayleigh random variable. Since this formula is not valid in the near field, in all the simulation results, we assume that *d* is greater than *d_o_*. In all the results, *d_o_* = 20m, *K_o_* = 50 and *β* = 3. The PU's protected distance *R_m_* is set to 10m. The secondary and primary users are uniformly distributed. All the simulations are performed using Monte Carlo runs.

For each PU, there is a PU protection area, wherein the strengths of the cognitive radio signals must be constrained. We define as *R*, the radius of the protected circular area for each individual PU. Given a distance, *d_m_*, between the SU base station and the mth PU and the radius, *R_m_*, of the protected circular area of the *m*th PU, the channel from the source to the *m*th PU in the *k*th SU band is given as:

(12) $$g_{m,k}^{s}=\frac{{\tilde{g}}_{m,k}^{s}}{{\left(d_{m}-R_{m}\right)}^{\beta }}$$

where ${\tilde{g}}_{m,k}^{s}$ is the small scale fading and *β* is the path loss exponent. For simplicity, throughout this paper and in simulation results, we assume that *R*_1_ = *R*_2_ = ⋯ = *R*_M_.

We compare the results of EDA and MEDA with the standard continuous genetic algorithm (GA) [38]. The EDA, MEDA and GA use IGS-CSSP and IGS-CSRA for constraint satisfaction. We will use the the word “non-green communication” when we set *w*_1_ = 1 and *w*_2_ = 0. In all the simulation results, we set the (M)EDA/GA parameters as (Δ_*t*_, *ρ_s_*, *I_Ter_*) = (20, 0.5, 1000), where Δ*_t_*, *ρ_s_*, *I_Ter_* are the population, selection probability and maximum iteration, respectively.

### 4.2. Throughput and CO_2_ Emissions Trade-Off

**Figure 4.**
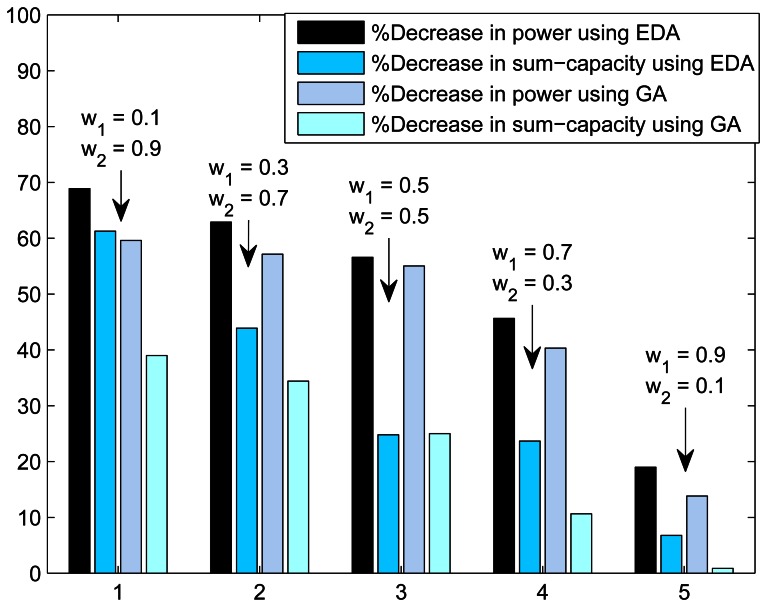
Power and sum-capacity trade-off with $\left(M,K,L,I_{mk}^{\text{max}}\right)=\left(1,10,20,1W\right)$.

In Figure 4, we present the trade-off plot of sum-capacity and total transmission power(or CO_2_ emissions). The trade-off is calculated between the green communication and without green communication. Trade-off is presented as the percentage decrease in sum-capacity and percentage decrease in power consumption. The decrease in sum-capacity and decrease in power consumption is calculated using the expressions:

(13) $$\begin{array}{cc} \text{Decrease in power} & =E^{CO_{2}}\;(w_{1}=1,w_{2}=0)-\\ & E^{CO_{2}}\;(w_{1}=x,w_{2}=1-x) \end{array}$$

(14) $$\begin{array}{cc} \text{Decrease in sum-capacity} & =\sum_{k}C_{k}\left(w_{1}=1,w_{2}=0\right)-\\ & \sum_{k}C_{k}\left(w_{1}=x,w_{2}=1-x\right) \end{array}$$

where 0 < *x* < 1. Figure 4 shows the effect of green communication by changing the values of weights *w*_1_ and *w*_2_. The results show that when *w*_2_ is more than *w*_1_, there is more reduction in CO_2_ emissions (percentage decrease in power). The reduction in CO_2_ emissions comes at the cost of throughput reduction. From the results, we can observe that CO_2_ emissions will decrease by 50- to 70-percent at the cost of 10- to 30-percent loss of throughput when *w*_2_ ≥ *w*_1_. This means that with little sacrifices in throughput, there is a big gain in power. The different weight settings are suitable for different geographical conditions and regulatory policies. The results also show that the performance of EDA is better than GA.

### 4.3. Performance of Proposed Schemes

**Figure 5.**
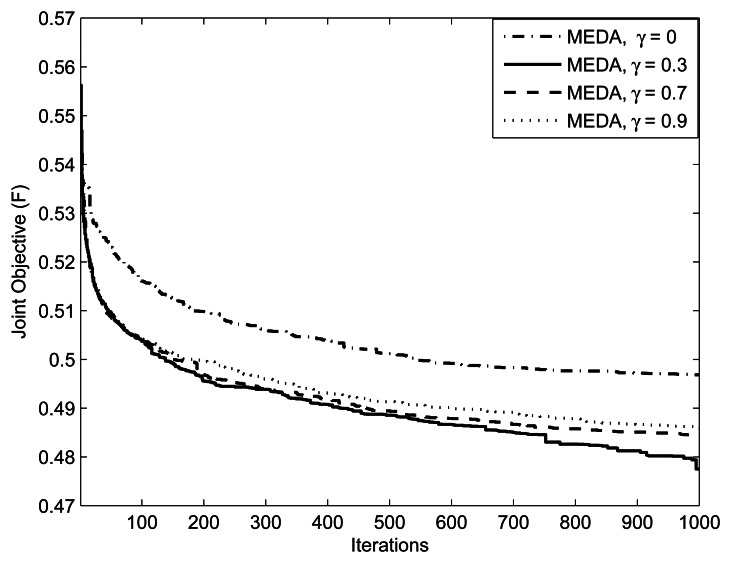
Effect of threshold parameter on EDA. The parameters are $\left(L,K,M,I_{m,k}^{\text{max}},w_{1},w_{2}\right)=\left(10,10,1,1W,0.5,0.5\right)$.

**Figure 6.**
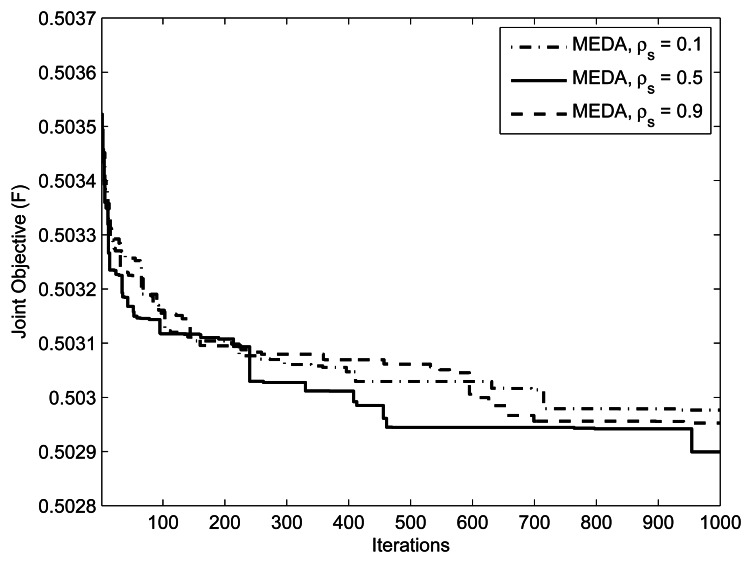
Effect of selection probability *ρ_s_* on EDA. The parameters are $\left(L,K,M,I_{m,k}^{\text{max}},w_{1},w_{2}\right)=\left(10,10,1,1W,0.5,0.5\right)$.

Figure 5 focuses on the method of applying thresholds on EDA, which is described in section 3.4. We ran an EDA with parameters $\left(M,I_{m,k}^{\text{max}},w_{1},w_{2},\delta \right)=\left(1,10mW,0.5,0.5,5\right)$ for threshold values = {0.3, 0.7, 0.9}. Note that setting *γ*= 1 is equivalent to not applying the threshold at all. Setting the threshold closer to 1 means that the algorithm generates the population from an almost identical distribution in each iteration; that is, the algorithm does not take advantage of the natural selection. An interesting issue is what values of the threshold facilitates the computation. From Figure 5, we can observe that the performance of EDA is poor at *γ* = 0.9 and 0.7. We can interpret this as, at the threshold values of *γ* = 0.9 and 0.7, which are close to 1.0, the algorithm does not evolve significantly. Figure 6 presents the effect of selection probability, *ρ_s_*, on the performance of EDA. The parameters are $\left(M,I_{m,k}^{\text{max}},w_{1},w_{2},\delta \right)=\left(1,10mW,0.5,0.5,5\right)$. From Figure 6, we can observe that the performance of EDA is better if selection probability, *ρ_s_*, is not close to either 0 or 1. There will be no evolution if selection probability, *ρ_s_*, is close to 1, the EDA will behave like a random algorithm. On the other hand, if selection probability, *ρ_s_*, is close to 0, the algorithm does not evolve significantly, because it is more likely that EDA will get stuck in the local optimum.

**Figure 7.**
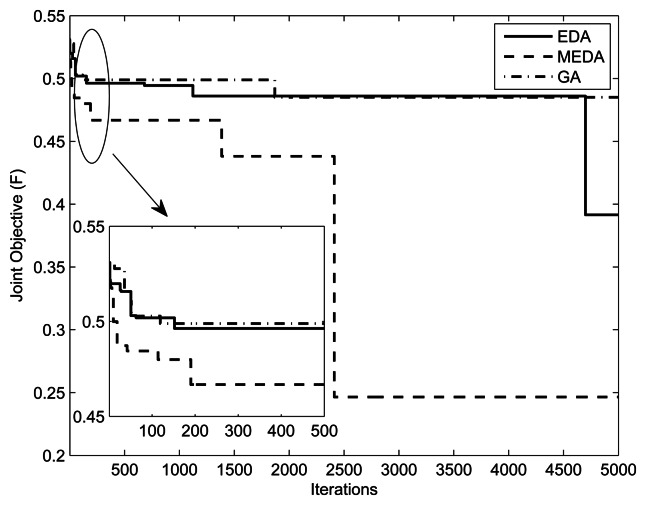
Iterations *vs.* Fitness plot with (*w*_1_, *w*_2_) = (0.5,0.5).

**Figure 8.**
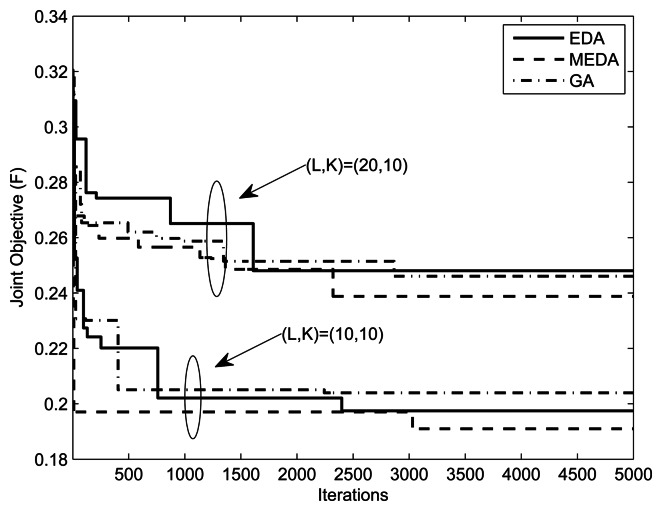
Iterations *vs*. Fitness plot with (*w*_1_, *w*_2_) = (0.1, 0.9).

Figures 7 and 8 present the fitness *vs.* iterations plot for different numbers of relays and users. The fitness function was defined in Equation (7). The parameters are $\left(M,I_{m,k}^{\text{max}},w_{1},w_{2},\delta \right)=\left(1,10mW,0.1,0.9,5\right)$ and (1, 10*mW*, 0.5, 0.5, 5), respectively. The value of threshold *γ* is set to 0.3. From the results, we can observe that the performance of MEDA is better than EDA and GA. This is because a simple EDA and GA can get stuck in the local optimum after a few iterations. We can also note that fitness values with less relays is better than fitness values with more relays. This is because the assignment takes more iterations for a large number of relays.

**Figure 9.**
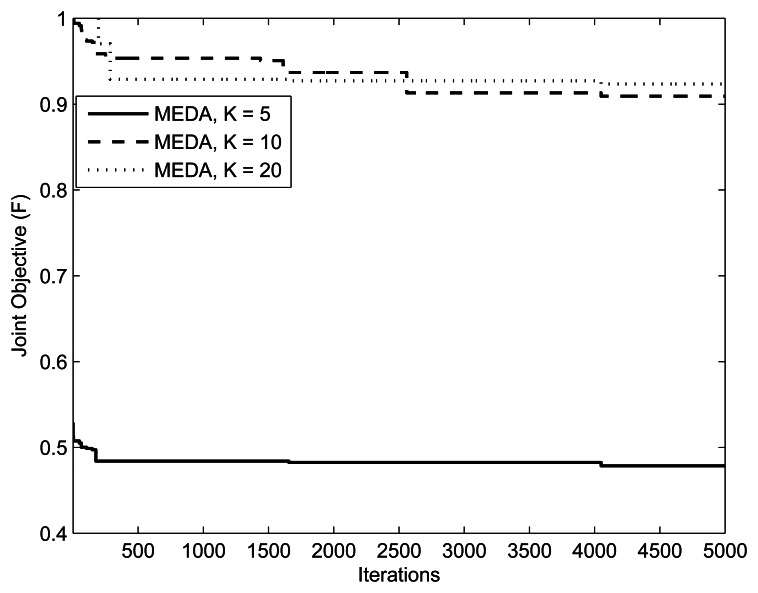
Iterations *vs*. Fitness plot with (*w*_1_, *w*_2_) = (0.5, 0.5).

**Figure 10.**
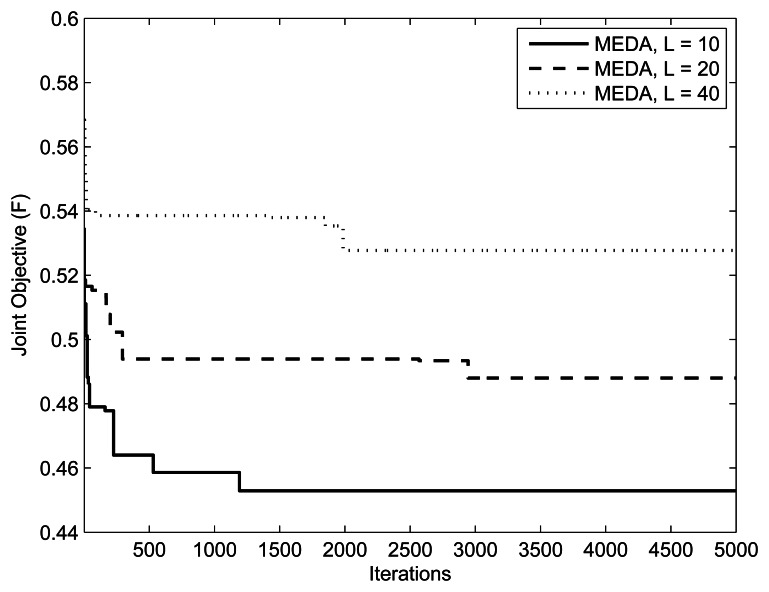
Iterations *vs*. Fitness plot with (*w*_1_, *w*_2_) = (0.5, 0.5).

**Figure 11.**
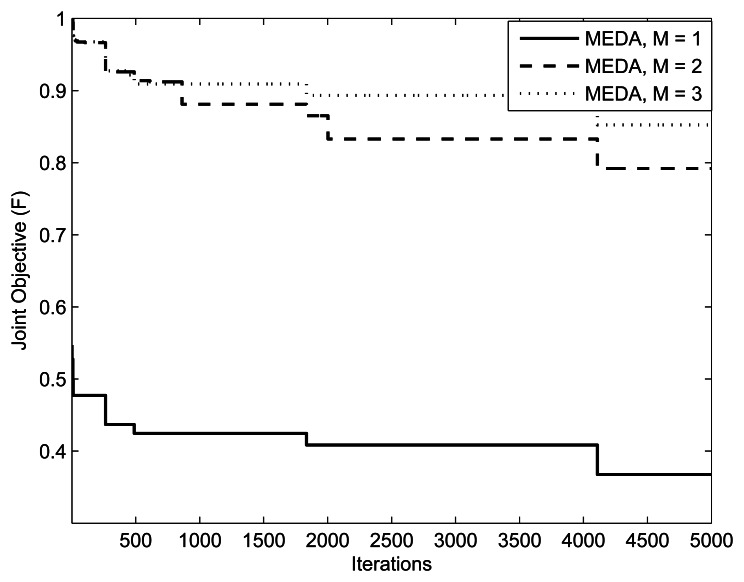
Iterations *vs*. Fitness plot with (*w*_1_, *w*_2_) = (0.5, 0.5).

Figures 9, 10 and 11 present the impact of SUs, relays and PUs on the performance of the green sensor network. The parameters are $\left(K,L,M,I_{m,k}^{\text{max}}\right)=\left(\{5,10,20\},10,1,10mW\right)$, (20, {10.20, 40}, 10*mW*) and (10,10,{1, 2, 3},10*mW)*, respectively. In all simulations, (, *w*_1_, *w*_2_, *δ*) are set to (0.5,0.5, 5). We observe that for a fixed number of secondary users, the fitness of the objective function will always be better with a lesser number of relays. This is because the assignment takes more iterations for a large number of relays. In the simulation results, we also observe that the objective function is better at a low number of PUs. This is because the relay assignment needs to satisfy more interference constraints as the number of PUs increases.

## 5. Conclusion

In this paper, we presented a multi-objective framework for resource allocation in green cooperative cognitive radio sensor networks. The estimation-of-distribution algorithm with iterative greedy scheme is used to solve the multi-objective optimization problem. The simple underlying concept and ease of implementation of the proposed algorithm make EDA a suitable candidate for green resource allocation.

## Figures and Tables

**Table 1.** Notations.

**Symbol**	**Definition**

*K*	Number of secondary users
*M*	Number of primary users
*L*	Number of relays
*I_m_*	Interference threshold at *m*th PU
$h_{l}^{s}$	Channel between the source and the *l*th relay
$h_{k}^{l}$	Channel between the *k*th SU and the *l*th relay
*p*_l_	Transmission power of the *l*th relay
$p_{l}^{\text{max}}$	Maximum power of the *l*th relay
$p_{k}^{s}$	Transmission power of the source at the *k*th SU band
*P_s_*	Maximum available power of the source
$g_{m,k}^{s}$	Channel between the source and the *m*th PU in the *k* SU band
${\text{g}}_{m,k}^{l}$	Channel between the *m*th PU and the lth relay in the *k* SU band
*ε*	binary assignment indicator
*F*()	Fitness function as mentioned in Equation (7)
*W_H_*	Upper limit of the EDA search window
*W_L_*	Lower limit of the EDA search window
Δ*_t_*	The population at the *t*th iteration and |Δ*_t_* | denotes the cardinality
*η_t_*	The set of best candidate solutions selected from set |Δ*_t_*| at the *t*th iteration.
*ρ_s_*	The selection probability. The EDA selects *ρ_s_*|Δ*_t_*| individuals from the set |Δ*_t_*| to make up the set *η_t_*.
*I_Ter_*	The maximum number of iterations

**Table 2.** Common Parameter Values.

**Parameters**	**Values**

$p_{l}^{\text{max}}$	1 Watts
*ρ_s_*	10 Watts
*γ*	0.2
Δ*_t_*	20
*η_t_*	10
*ρ_s_*	0.5
I*_Ter_*	5000
